# Deep neural network model of haptic saliency

**DOI:** 10.1038/s41598-020-80675-6

**Published:** 2021-01-14

**Authors:** Anna Metzger, Matteo Toscani, Arash Akbarinia, Matteo Valsecchi, Knut Drewing

**Affiliations:** 1 Justus-Liebig University Giessen, 35394 Giessen, Germany; 2 University of Bologna, 40126 Bologna, Italy

**Keywords:** Sensorimotor processing, Sensory processing, Attention, Perception, Network models, Motor control, Somatosensory system, Touch receptors, Human behaviour

## Abstract

Haptic exploration usually involves stereotypical systematic movements that are adapted to the task. Here we tested whether exploration movements are also driven by physical stimulus features. We designed haptic stimuli, whose surface relief varied locally in spatial frequency, height, orientation, and anisotropy. In Experiment 1, participants subsequently explored two stimuli in order to decide whether they were same or different. We trained a variational autoencoder to predict the spatial distribution of touch duration from the surface relief of the haptic stimuli. The model successfully predicted where participants touched the stimuli. It could also predict participants’ touch distribution from the stimulus’ surface relief when tested with two new groups of participants, who performed a different task (Exp. 2) or explored different stimuli (Exp. 3). We further generated a large number of virtual surface reliefs (uniformly expressing a certain combination of features) and correlated the model’s responses with stimulus properties to understand the model’s preferences in order to infer which stimulus features were preferentially touched by participants. Our results indicate that haptic exploratory behavior is to some extent driven by the physical features of the stimuli, with e.g. edge-like structures, vertical and horizontal patterns, and rough regions being explored in more detail.

## Introduction

The sense of touch substantially enriches our perception of the world. Touch sensations depend on active hand movements^1^. These exploratory movements are usually highly stereotypical, repetitive and depend on what information about an object is desired (*Exploratory Procedures*^2,3^). For instance we move the hand laterally over the object’s surface to judge its roughness, but keep it flat and static on top of it in order to judge the object’s temperature^2^. Also in haptic search on a 2D plane (e.g. for a certain raised line symbol) systematic movements are observed, such as parallel sweeps, zigzags, spirals^4,5^ or movements parallel to the outline of the search space^6^. Systematic exploration of an object’s surface is a sensible strategy for a perceptual system that cannot access a large portion of the stimulus at once such as touch. However, exploration in haptic search is less systematic when the whole hand can be used as compared to one finger^4,5^, suggesting that with broader sensory input stimulus-driven strategies can complement or even replace systematic movement as a more efficient approach. In line with this view, we found evidence for foveation-like behavior in whole-hand haptic search: humans perform a first quick and coarse exploration of the search space in which all the fingers and the hand are involved and then further explore in detail with the index and the middle finger only the parts of the stimulus which are more likely to be the target^6–8^. Importantly, detailed analysis was very prominent in the case it was associated with information gain and reduced when the target was easy to detect^8^, consistent with foveation-like behavior similar as in vision. These results imply that haptic exploration is not only based on systematic movements but also consists of local detailed analysis.

If haptic exploration is not solely systematic but includes detailed analysis, it can be expected that some object parts are more likely to be analyzed in depth than others, i.e. touch behavior can be predicted from physical stimulus properties. We provided preliminary evidence that local object features can drive touch exploration^9^. Using a linear regression model we could predict better than chance but to a little extent the spatial distribution of touch duration when participants touched ten 3D printed rigid plates with locally varying surface relief. The model used as predictors the spatial distributions of stimulus’ properties (relief height, spatial frequency, anisotropy and orientation) as they were defined to generate their surface relief. Thus, this model could therefore not directly relate exploratory behavior to the physical surface relief, i.e. exploratory behavior was not *stimulus-computable* (the model cannot be applied to different stimuli e.g. natural surfaces). Additionally, any non-linear relationship, such as tuning to specific feature values, which is very common in perceptual systems (e.g. orientation tuning^10^) was by definition disregarded by this model. Here we instead use state of the art deep learning techniques to predict touch behavior based on local physical properties of rigid haptic textures. Our present results prove that active touch not only consists of stereotypical stimulus-independent movements, but it is also driven by local salient features. Results generalize to different participants, performing a different task (Exp. 2) and exploring different stimuli (Exp.3).

**Figure 1:**
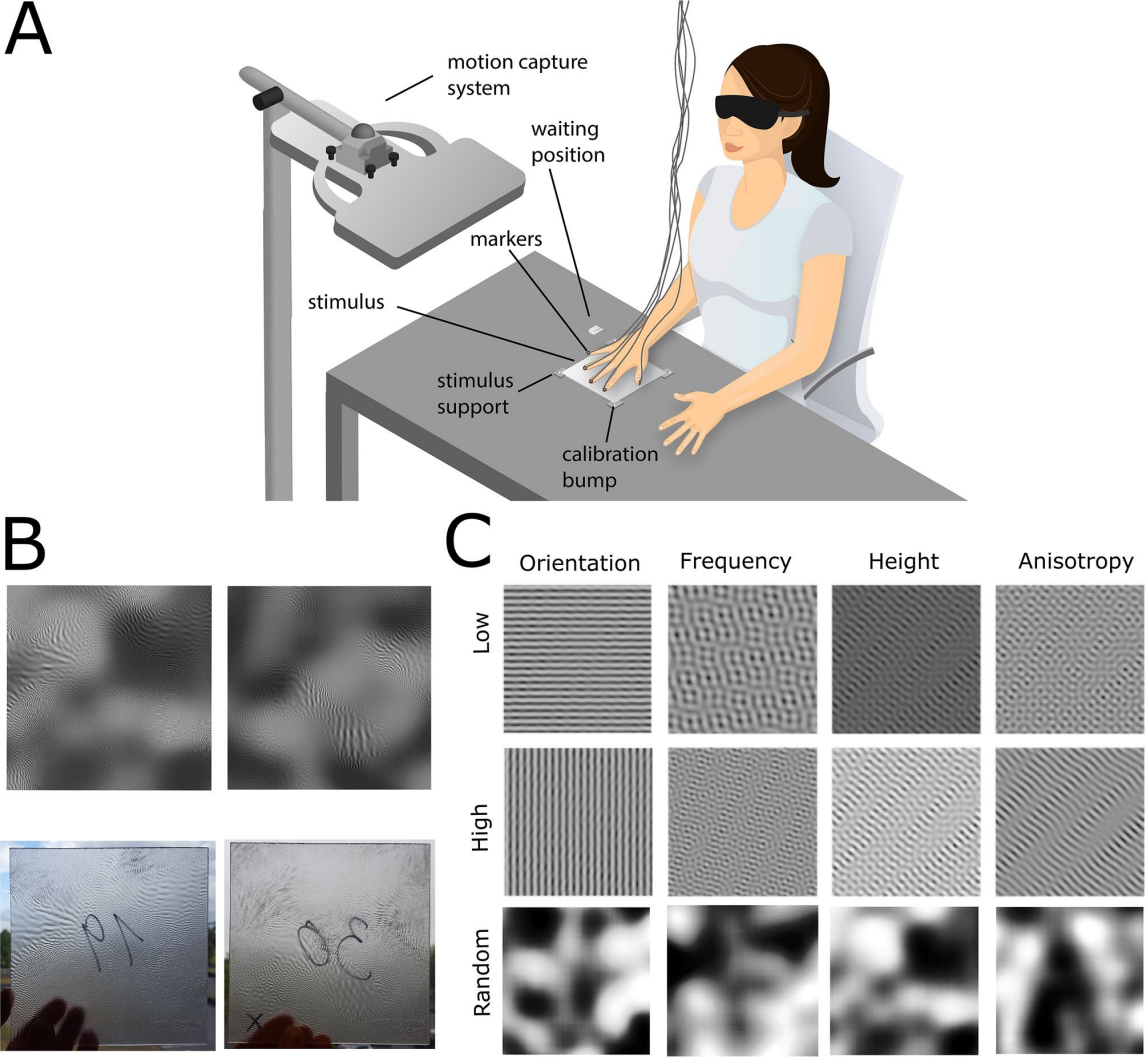
(**A**) Setup. Participants sat at a table together with the experimenter who positioned the stimuli in front of them. Stimuli were stabilized at the four corners. The position of each finger of the participant’s right hand was tracked with the ultrasound based motion tracking system (Zebris). Prior to the experiment the position of the right index finger was calibrated using the calibration bumps in the stimulus support corners. Between trials participants moved the hand to the waiting position marked as a finger-holder for the middle finger. (**B**) Example stimuli. Surface reliefs on top with their printed versions below. (**C**) Stimulus features used to create the stimuli: orientation direction (Orientation), spatial frequency (Frequency), surface relief height (Height) and spatial anisotropy (Anisotropy). The features are exemplified by uniform stimuli (not used in the experiment), in which all features are kept constant but one is set either to the minimum (“Low”, first row) or the maximum (“High”, second row) of the corresponding feature range. The third row (“Random”) shows four pink noise maps, one per feature, used to generate the example stimulus 19 in **B**).

In Experiment 1 twelve participants had to decide whether two rigid haptic surfaces were the same or different after subsequent blind-folded exploration with the right hand. They sat at a table and the position of each finger of the right hand was tracked at 50 Hz with an ultrasound based motion tracking system (Fig. 1A). The experimenter placed the stimuli in front of the participants one after the other. Exploration of each stimulus was not limited in time.

The stimuli were generated with the same algorithm as the ones in^9^. We printed with a 3D printer 60 new rigid plates (13.97 × 13.97 × 0.3 cm) with varying surface relief. The surface reliefs were created to offer a rich variability of texture features. They were first generated as 2D-images and then translated into 3D models by interpreting pixel intensity as depth (Fig. 1B). We defined the surface relief for each stimulus locally by independent random distributions of four features: relief height, spatial frequency, orientation and anisotropy, the values of which were coded between 0 and 1 (Fig. 1C, third row). For each feature the local code was then mapped to a value in the predefined feature meaningful range. For instance, the [0 1] interval of local codes was mapped to the distribution of relief height that ranged between 0.1 and 0.3 cm, while in the case of orientation values the mapping was to the range between 0° and 90°. Anisotropy defined whether stimulus modulations were locally rather elongated or blob like. Features are exemplified in Fig. 1C by two uniform stimuli in which all features are kept constant and one is set either to the minimum or the maximum value. To combine the features in one stimulus, uniform noise (randomly generated for each stimulus) was filtered in the frequency domain with an oriented band-pass filter (combination of a Gaussian and von Mises filters), the parameters of which were taken for every pixel from the distributions of the four features (Fig. 1C, last row). Exploratory behavior of the first stimulus was quantified as touch duration for each position (~ 0.5 mm spatial resolution) of the stimulus. Touch duration was computed only for the index and the middle finger, because these fingers are mostly involved in fine analysis of the stimulus^6–8^.

**Figure 2:**
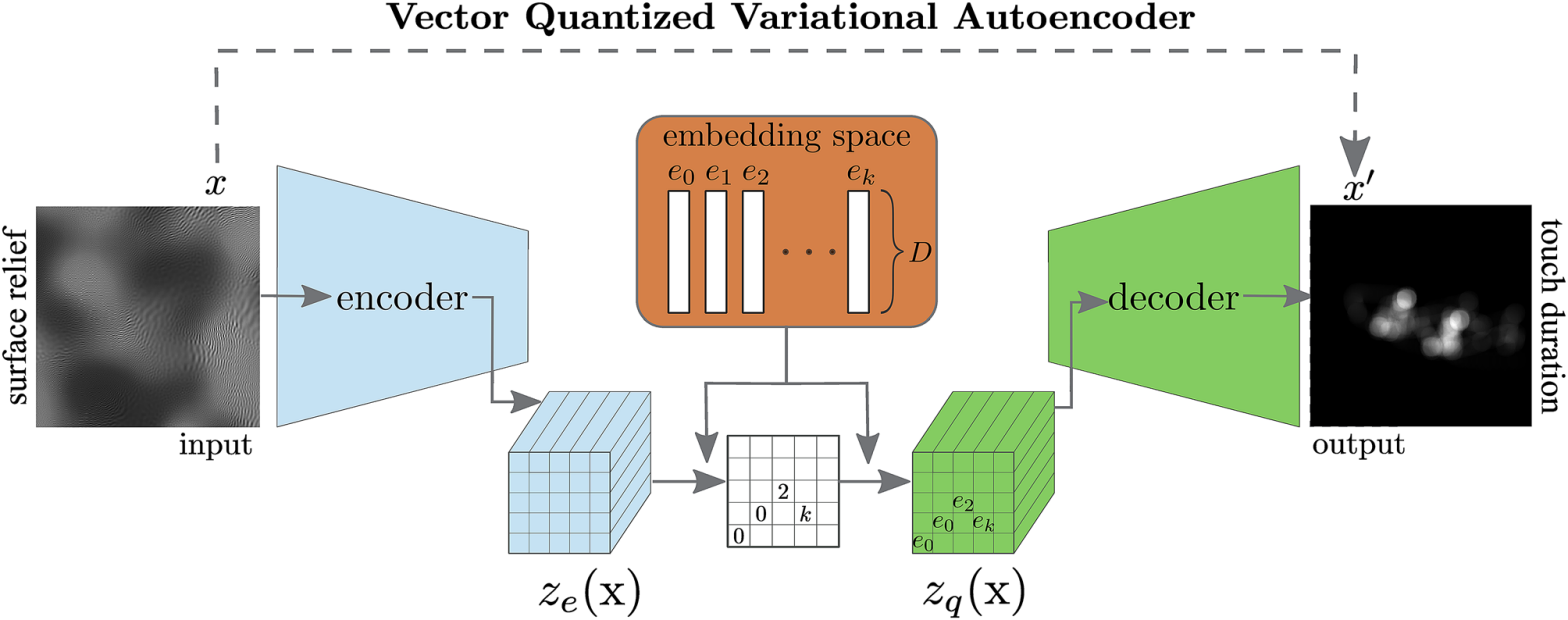
Schematic view of the model (VQ-VAE). The encoder is input with a surface relief map (one channel image) processing it to *z*_*e*_*(x)* after a couple of convolutional layers. The embedding space quantizes each point of *z*_*e*_*(x)* with the closest vector *e*_*k*_ to map it onto *z*_*q*_*(x)*. The decoder processes this new representation to generate output corresponding to touch duration (one-channel image).

We used Vector Quantized Variational Autoencoder: VQ-VAE^11^ to predict touch duration based on the surface relief of the haptic surfaces. Autoencoders are actively used for a large number of computer vision tasks with no restriction on the relation between input and output, for instance, edge detection^12^, image segmentation^13^, reconstruction of fMRI stimuli^14^ or image classification^15^. VQ-VAE is a type of deep generative model aiming to efficiently learn input signals by compressing its representation into a quantized latent space (see Fig. 2). This discrete nature of embedding space facilitates the interpretation of the network’s complex representation. The input to this model is the map of the surface relief (a 2D matrix whose pixel values vary in the range of 0 to 1 corresponding to smallest and largest surface elevation). The output is the local touch duration (i.e. how long each portion of the stimulus was touched for) obtained from participants who performed the task. VQ-VAE consists of three major components. (1) an encoder that processes the input data *x* to *z*_*e*_*(x)*; (2) a latent embedding space $\left\{e\right\}\in {R}^{K\times D}$, with *K* vectors of dimensionality *D*, that maps *z*_*e*_*(x)* onto *z*_*q*_*(x)* by identifying the nearest vector *e*_*i*_ to *z*_*e*_*(x)*; (3) a decoder that reconstructs the final output *x’* with a distribution $p\left(x\vee {z}_{q}\left(x\right)\right)$ over the input data. The encoder is essentially a number of convolutional layers and the decoder is a number of deconvolutional layers. The objective function the network optimizes is a simple mean squared error (squared L2 norm) between the actual touch duration and model’s prediction. The final model, used for evaluation and characterization in following analysis, was trained on 80% of the data randomly sampled from all participants and all trials, and tested on the remaining 20%. In addition, to ensure our findings generalize well, we performed a standard *n*-fold cross-validation procedure by excluding the data of one participant for testing and using the rest for training. Effectively, for the cross-validation we trained twelve models of an identical architecture.

We used a ROC analysis^16^ to assess the performance of the model and compare it with the predictions based on the mere surface relief of the stimuli (i.e. how well relief height varying between 1 and 3 mm can discriminate between low and high touch duration). To investigate what the DNN model has learned we correlated its responses to the local features of a large number of simulated haptic textures similar to the ones participants explored. Finally, we repeated the ROC analysis for each of the 12 n-fold cross-validation models, to test how well they could predict touch behavior of participants excluded from training. This allowed us to check whether our main results were due to over-fitting. In Experiments 2 and 3, different sets of participants (10 and 8, respectively) explored every stimulus without being asked for a particular task. In Experiment 2 we used the same stimuli as in Experiment 1 and in Experiment 3 participants explored in each trial one of four 3D printed natural surfaces (cliff rocks, pebbles, ground soil and cracked earth) generated from 3D scans (textures.com). The model trained with the data from Experiment 1 was used to predict touch behavior in the other two Experiments. This allowed to test the generality of our findings across tasks, stimuli and participants.

## Results

**Figure 3:**
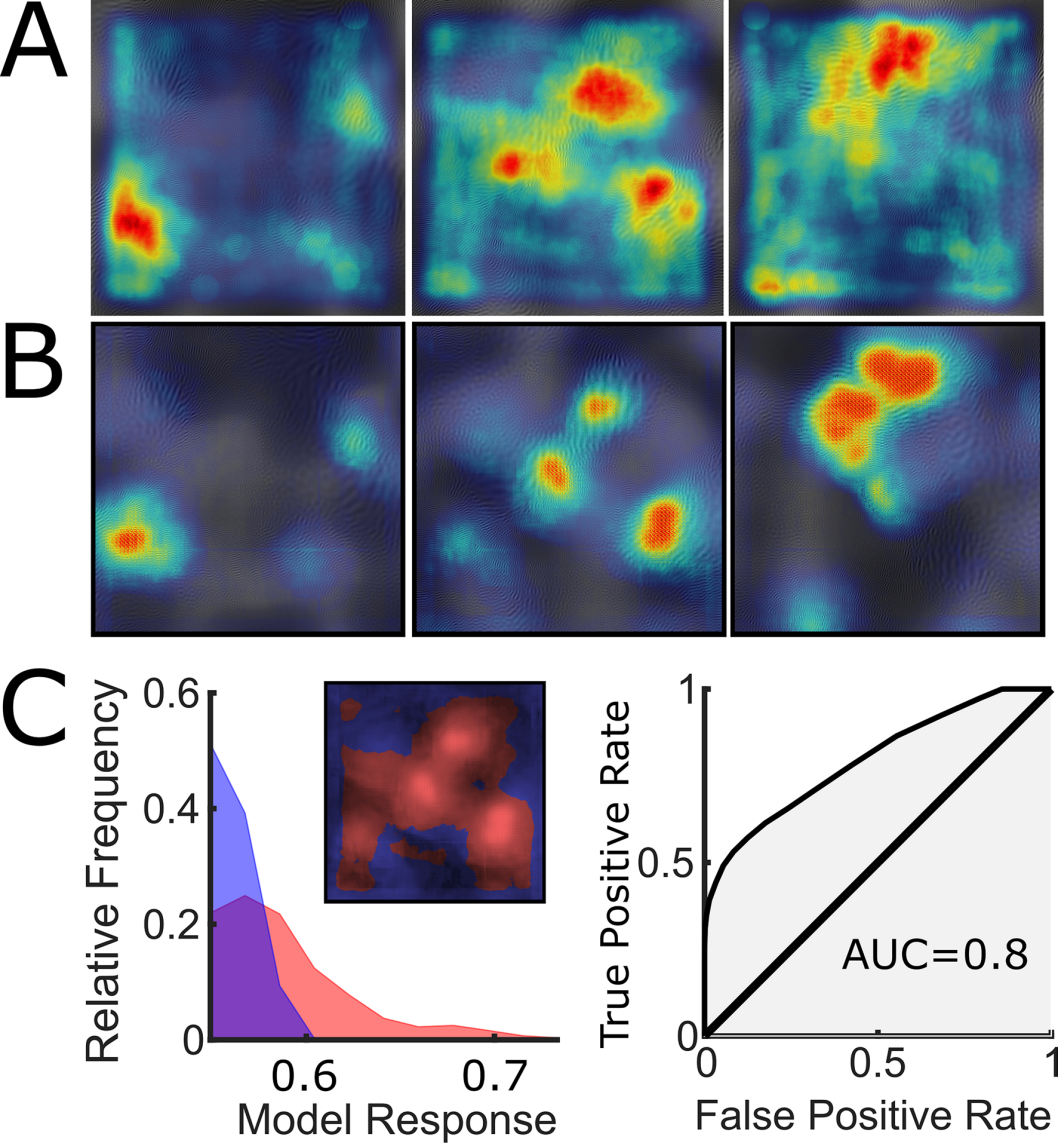
Model’s predictions. (**A**) Touch duration averaged across trials and participants for three example stimuli. (**B**) Model Predictions for the three stimuli. Each prediction corresponds to the stimulus above. Red represents high and blue low touch duration. The heat maps are depicted on top of the surface relief maps. To express model’s prediction the eight-bit output is normalized so that the maximum value corresponds to one. The minimum output value did not correspond to zero as predictions ranged between ~ 0.56 and 1. (**C**) Example of ROC analysis for the middle example stimulus in A&B. Left panel: the icon on the top right represents how the stimulus surface is split depending on touch duration. Red areas represent the half of the surface with higher touch duration. Blue areas the half with lower touch duration. Model’s predictions are represented by intensity: lighter areas correspond to predicted high touch duration and darker area to predicted low touch duration. The red distribution in the histogram represents the relative frequency (*y*-axis) of the model’s prediction corresponding to the high touch duration half of the stimulus (red area in the icon). The blue corresponds to the low touch duration. Right panel: ROC curve corresponding to the low touch duration and the high touch duration distributions represented by the histograms in the left panel.

Figure 3 shows touch duration averaged across participants (heat maps, Fig. 3A) and the model’s predictions (heat maps, Fig. 3B) for three example stimuli. The predictions seem very similar to the actual touch duration. This impression is confirmed by the example ROC (Receiver operating characteristic) analysis in Fig. 3C. The surface of the stimulus is divided into most (red area in Fig. 3C icon) and least touched (blue area in Fig. 3C icon) portions depending on the touch duration distribution. If the model is predictive for touch duration, the distribution of the model’s response within the most touched portion should be higher than within the least touched one, as shown by the histogram in Fig. 3C (most touched in red, least in blue). The area under the ROC curve (right panel in Fig. 3C) is a criterion independent measure of discrimination between the two distributions, ranging from overlapping (AUC = 0.5) to completely separated (AUC = 1). Thus, AUC is a measure of the predictors’ performance. The distributions in the example are fairly separable (AUC = 0.8), indicating that the model can predict average touch duration for that stimulus.

For this analysis the stimulus surface was split in two equal parts: one corresponding to higher and one to the lower touch duration (i.e. most and least touched half of the stimulus). This is an arbitrary choice, as less than half of the touched locations can be used to define the least and the most touched portions (e.g. the 10% of the stimulus area could be selected, corresponding to the shortest touch duration for the least touched portion and the longest for the most touched one). To check whether results depend on this choice, we conducted the following analysis with different selection criteria in the range between 5% and 50%.

We repeated the ROC analysis on all the trials and averaged AUCs across trials and participants. In order to have quantitative comparisons for the model’s performance, the ROC analysis was also conducted with the mere surface relief of the stimuli *(Relief)* as a predictor. We also assessed the performance of touch duration averaged across trials and participants (*Average Touch*) and predicted touch duration for each map and participant with the average touch duration of other participants exploring the same map (‘*Gold Standard’*). The latter measure indicates the inter-subject consistency and with it the upper limit for performance. Because touch exploration is known to be dominated by stereotypical movements independent of the local features of the stimuli (e.g. orientation movements parallel to the stimulus edges^6^), we expected *Average Touch* to be highly predictive for the observed touch behavior.

**Figure 4:**
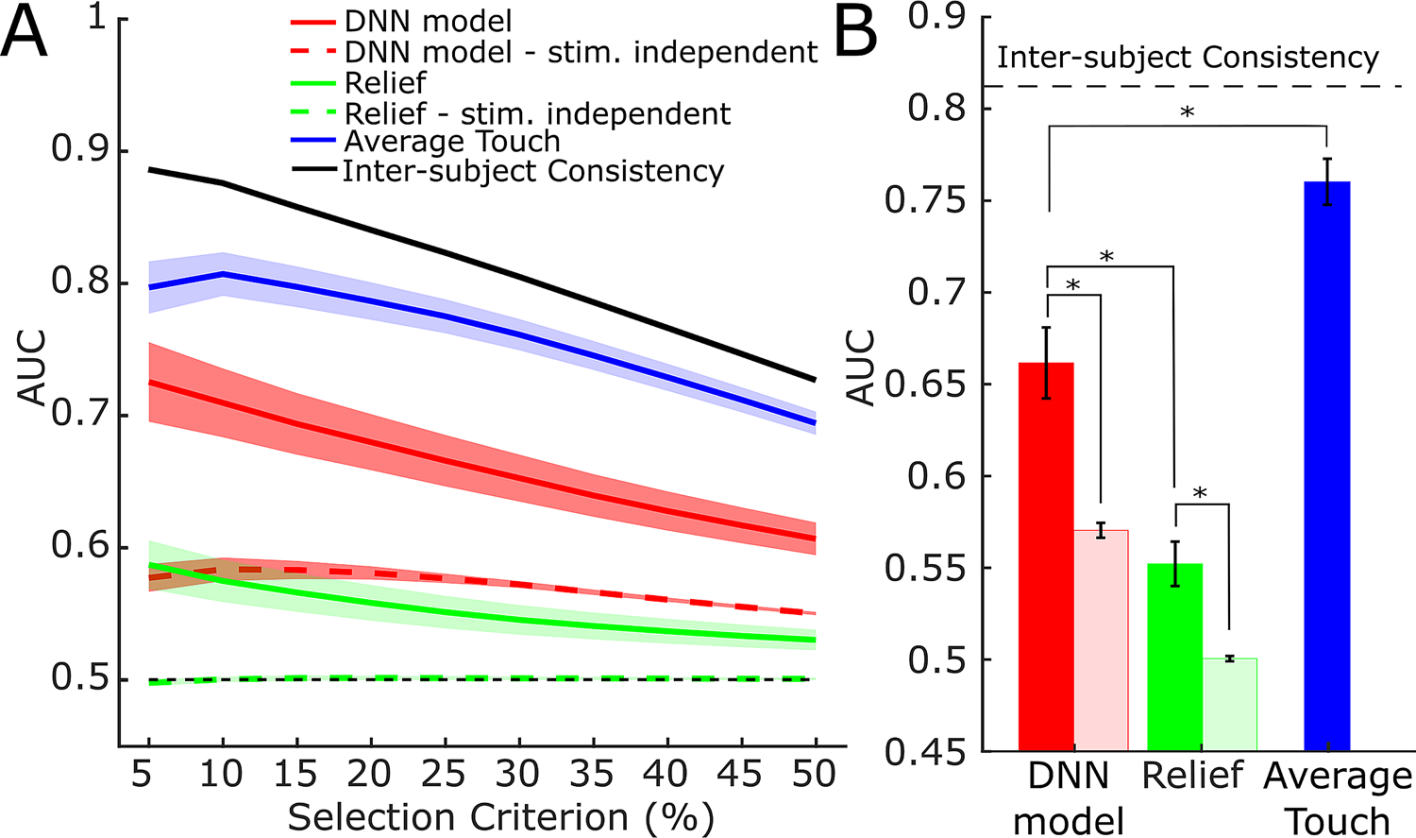
ROC analysis. (**A**) AUC (*y*-axis) computed with different selection criteria (*x*-axis). AUCs for different predictors are depicted with different colors, as indicated in the legend. Continuous lines represent the performance based on predictors computed for the stimuli that were actually explored in every trial. Dashed lines represent the stimulus independent AUCs. AUCs are averaged across participants; the colored areas represent the standard error of the mean. (**B**) AUC averaged across selection criteria. Faint colors indicate AUC based on stimulus-independent predictions. Error bars represent the standard error of the mean across participants. Significant comparisons are marked with *, indicating p < 0.0005.

Figure 4 shows the results of the ROC analysis for the *DNN Model* prediction (red), for *Relief* (green) and for the A*verage Touch* (blue). Inter-subject consistency is indicated in black. AUCs (on the *y*-axis) are computed with different selection criteria (*x*-axis). Continuous lines represent performance of the predictors. For each predictor we also computed the AUC using for each trial all the maps that were not presented in that trial (dashed lines). This measure of stimulus-independent performance is a crucial control to understand how much predictions depend on the stimulus local features rather than on stimulus independent tendencies of the participants to prefer certain regions to others.

In general, performance is higher with lower selection criteria, this is probably because the two distributions individuated by the more conservative criteria are more distinguishable. AUCs for the *DNN Model* (red line) are always above 0.5 indicating that the most and least touched regions can be discriminated based on the model’s predictions for all selection criteria. Also, AUCs are always higher when based on stimulus-specific predictions (red continuous line vs. red dashed line). Crucially, this proves that touch behavior is based—at least in part—on stimulus features. However, the red dashed line is also above 0.5, indicating that the model has learned some map-independent component of the touch behavior.

The green lines indicate performance of the mere surface relief. Performance is clearly lower than the DNN model’s, but different from 0.5, when stimulus-specific. When the association between stimuli and trials is broken (i.e. stimulus-independent), *Relief* shows no predictive power (green dashed line sits on 0.5), as the surface relief is uncorrelated between the different stimuli.

Finally, *Average Touch* has an exceeding predictive power (blue line). This is consistent with the knowledge that touch behavior is highly stereotypical.

For statistical testing we averaged across selection criteria and used *t*-tests on individual values to compare between stimulus-specific and stimulus-independent performance, for each model. *t*-tests were also used to compare the performance of the *DNN Model* to the one of the other predictors. Average AUCs are shown in Fig. 4B. The *DNN Model* performs significantly better than the *Relief, t*(11) = 11.25, *p* < 0.0005 but worse than *Average Touch, t*(11) = − 6.56, *p* < 0.0005. *DNN model* performs better than its stimulus-independent predictions, *t*(11) = 5.37, *p* < 0.0005, indicating that the model has learned to predict touch behavior based on stimulus properties. This is true also for *Relief*, *t*(11) = 4.49, *p* < 0.001. As stimulus-independent surface relief has no predictive power by definition, this latter test only reveals that *Relief* can significantly predict touch behavior.

**Figure 5:**
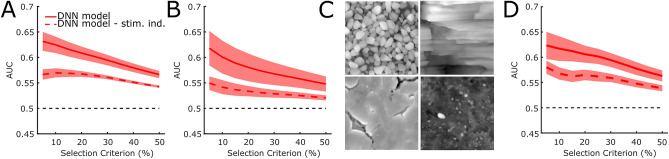
(**A**) Cross-validation (**B**) Generalization to free exploration. (**C**) Natural stimuli used in Experiment 3. (**D**) Generalization to natural textures. AUC (y-axis) computed with different selection criteria (x-axis). The lines represent the average across different models. Filled areas represent the standard error of the mean. Continuous lines represent the AUC based on the stimulus specific predictions; dashed line on the stimulus independent predictions.

In order to rule out that the feature-based predictive power of the model is due to over-fitting, we cross-validated our results. To do so, we iteratively excluded one participant from our ground-truth data, and trained the model on the remaining. Then, we repeated the AUC analysis on the participant we left out. We therefore trained one model per participant on the data of the other participants. Figure 5A shows the AUC averaged across models. Performance is significantly better when predictions are stimulus-specific, as indicated by a *t*-test on AUCs averaged across selection criteria, *t*(11) = 4.96, *p* < 0.0005.

Also, we investigated whether our *DNN model* is general enough to predict touch behavior beyond the task and the specific stimuli we involved participants with in Experiment 1. Thus, we performed two new experiments. In Experiment 2 we asked a new set of participants (N = 10) to freely explore the stimuli used in Experiment 1 (*“Please try to use your sense of touch to understand what kind of object is in front of you.”*) and recorded touch duration. For Experiment 3 we generated four new stimuli by 3D printing 3D scanned natural surfaces (cliff rocks, pebbles, cracked earth and ground soil, Fig. 5C, textures.com) and let yet another set of participants (N = 8) freely explore them. Then we used the *DNN model* trained on the original data to predict the new data-sets. For both Experiments the ROC analysis revealed that the model can predict touch behavior based on local features, i.e. AUC averaged across selection criteria is higher when stimulus-specific (Fig. 5B,D, Exp.2: *t*(9) = 2.53, *p* = 0.028, Exp.3: *t*(7) = 3.25 *p* = 0.014).

**Figure 6:**
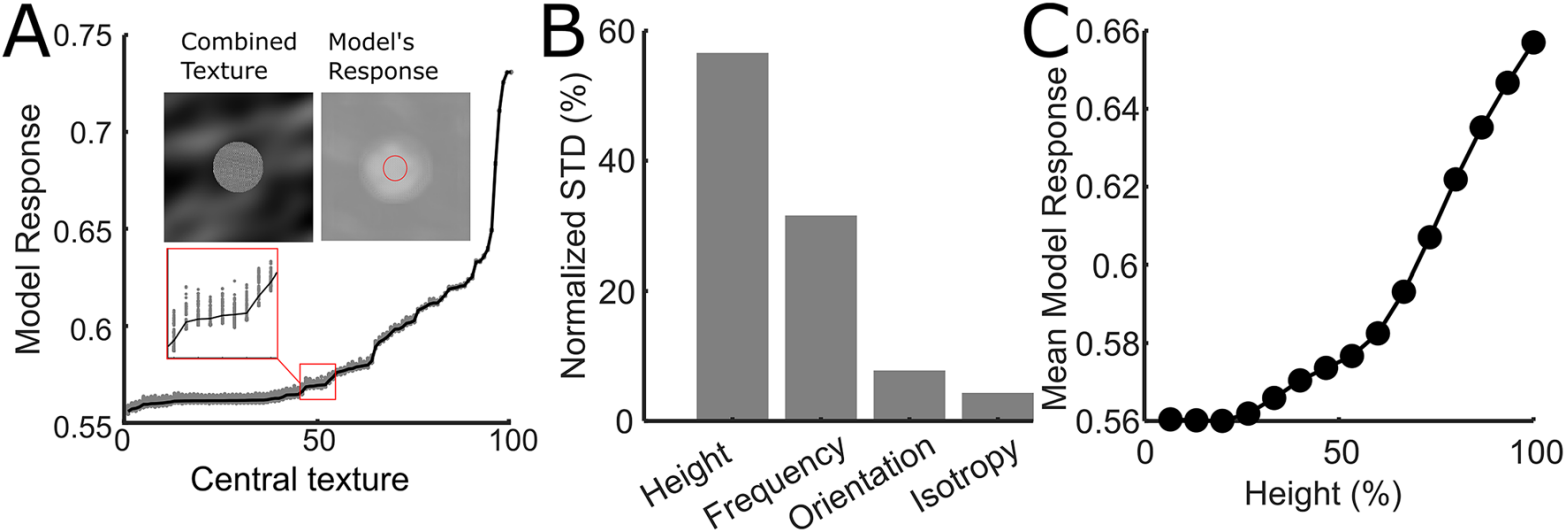
Model’s response. (**A**) Model response to combined textures. The icons illustrate an example of combined texture (left) and the associated model response. Model responses (gray dots) averaged across a finger–sized circle in the center are plotted (*y*-axis) against the 100 different central textures (*x*-axis). The central textures are sorted according to the model response averaged across their surrounds (black line). (**B**) Variability of model response to the uniform textures across feature levels. Standard deviation on the *y*-axis for the different features, on the *x*-axis. The standard deviation is normalized to sum to 1 over the 4 features and expressed in percentage. (**C**) Mean Model response (*y*-axis) as a function of the height feature (*x*-axis), expressed as percentage of the maximum relief.

Results prove that touch behavior is at least in part driven by local stimulus properties, as they are captured by the *DNN model*. It is therefore sensible to investigate what are the stimulus properties preferred by the model, as they likely represent what drives human touch behavior. To do so, we generated a large number of uniform textures (like in Fig. 1C—first two rows from top) and correlated the model’s responses with the stimulus properties. Each uniform texture was identified by a combination of four values, one for each of the features used to generate our stimuli (see Fig. 1C). Because we assumed that the model’s responses are local, i.e. the responses to the local properties of a portion of the stimulus area are not influenced by its surround, we averaged model’s response to each uniform texture and correlated it with the feature values of that texture. To check this assumption, we performed the following control: We analyzed the model’s responses to combined textures (see icons in Fig. 6A) consisting of the center from one uniform texture and the surround from other uniform textures. Crucially, each of 100 centers was associated with 100 different surrounds, for a total of 10,000 combined textures. Figure 6A shows that the model’s response (*y*-axis), averaged within a finger-sized central region (Fig. 6A, red circle), only depends on the center (*x*-axis) and does not vary much with different surrounds (different gray dots for each value along the *x*-axis). This corroborates the assumption that model’s responses are local.

Figure 6B shows the variability in model response to the uniform textures, averaged across the levels of each feature, for each combination of the other features and averaged across these combinations. This is a measure of how much each feature drives model’s responses. The model mostly changes response with changes of *Height*, but also differences in spatial frequency play a major role. *Orientation* and *Anisotropy* can modulate the model response, but to a lesser extent. The monotonic relationship between *Height* and model’s response is shown in Fig. 6C.

In order to describe the tuning of the model to *Frequency* and *Orientation*, we expressed the model responses as percentile ranks relative to the responses to the whole data-set of uniform textures, which gives a direct measure of the model’s preference.

**Figure 7:**
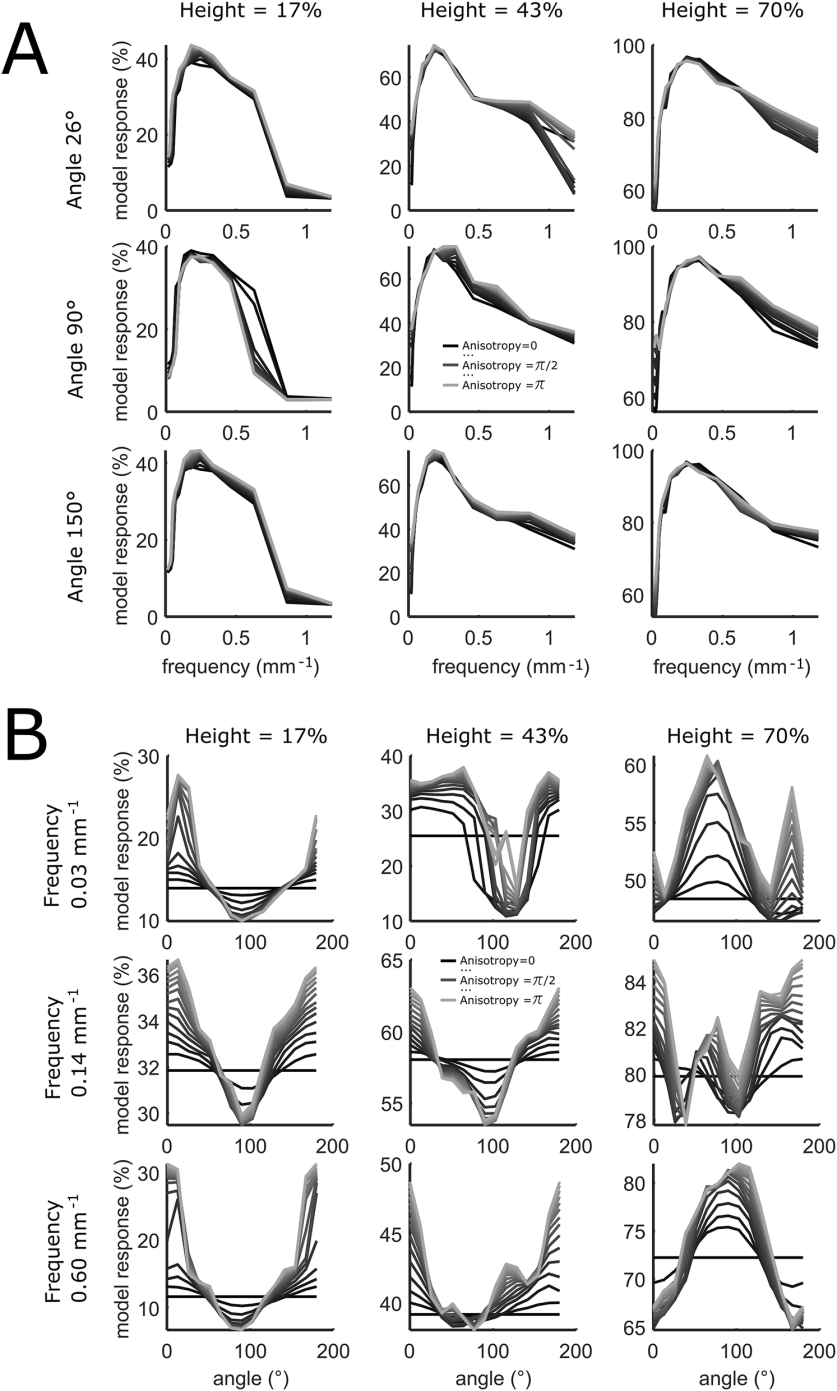
(**A**) Frequency tuning. Percentile Ranks of model response (*y*-axis) as a function of Frequency (*x*-axis). The different levels of Height and Orientation are binned into three bins corresponding to the different panels. Panels from left to right indicate different heights, from top to down different orientations. Lighter lines indicate higher anisotropy. (**B**) Orientation tuning. Percentile Ranks of model response (*y*-axis) as a function of orientation angle (*x*-axis). The different levels of Height and Frequency are binned into three bins corresponding to the different panels. Panels from left to right indicate different heights, from top to down different frequencies. Lighter lines indicate higher anisotropy, which varies from black (0) to lightest gray (π), as indicated in the legend.

Figure 7A shows the percentile rank of the model responses (model responses (%)) as a function of *Frequency*, for different levels of *Height*, *Anisotropy* and *Orientation*. The responses profile suggest frequency tuning, with a response peak around 0.2/0.3 cycles per millimeter, irrespective of *Height*, *Orientation* and *Anisotropy*. Figure 7B shows the percentile rank of the model responses as a function of *angle* for different levels of *Height*, *Anisotropy* and *Frequency*. Responses suggest a tuning profile that changes with *Height.* Namely, for small *Heights* the model responds most to horizontal angles (~ 0°); for higher *Heights* to vertical (~ 90°), irrespective of *Frequency*. Orientation tuning is more pronounced with high anisotropy, as low anisotropy implies a blobby pattern with no orientation. Thus, the model seems to be tuned to cardinal directions. However, the preference of one or the other cardinal direction changes with *Height*. Overall, higher responses are associated with higher *Anisotropy* (represented by the color of the lines, from dark to light for low to high anisotropy, respectively). This suggests a preference for lines rather than blobs.

For a different analysis, we generated 2000 random textures like the ones explored by the human participants, fed them into the model and reversed-correlated the local feature values with the model’s response. With this analysis we did not systematically vary the feature values, but the stimuli were as complex as the ones used to train the model.

**Figure 8:**
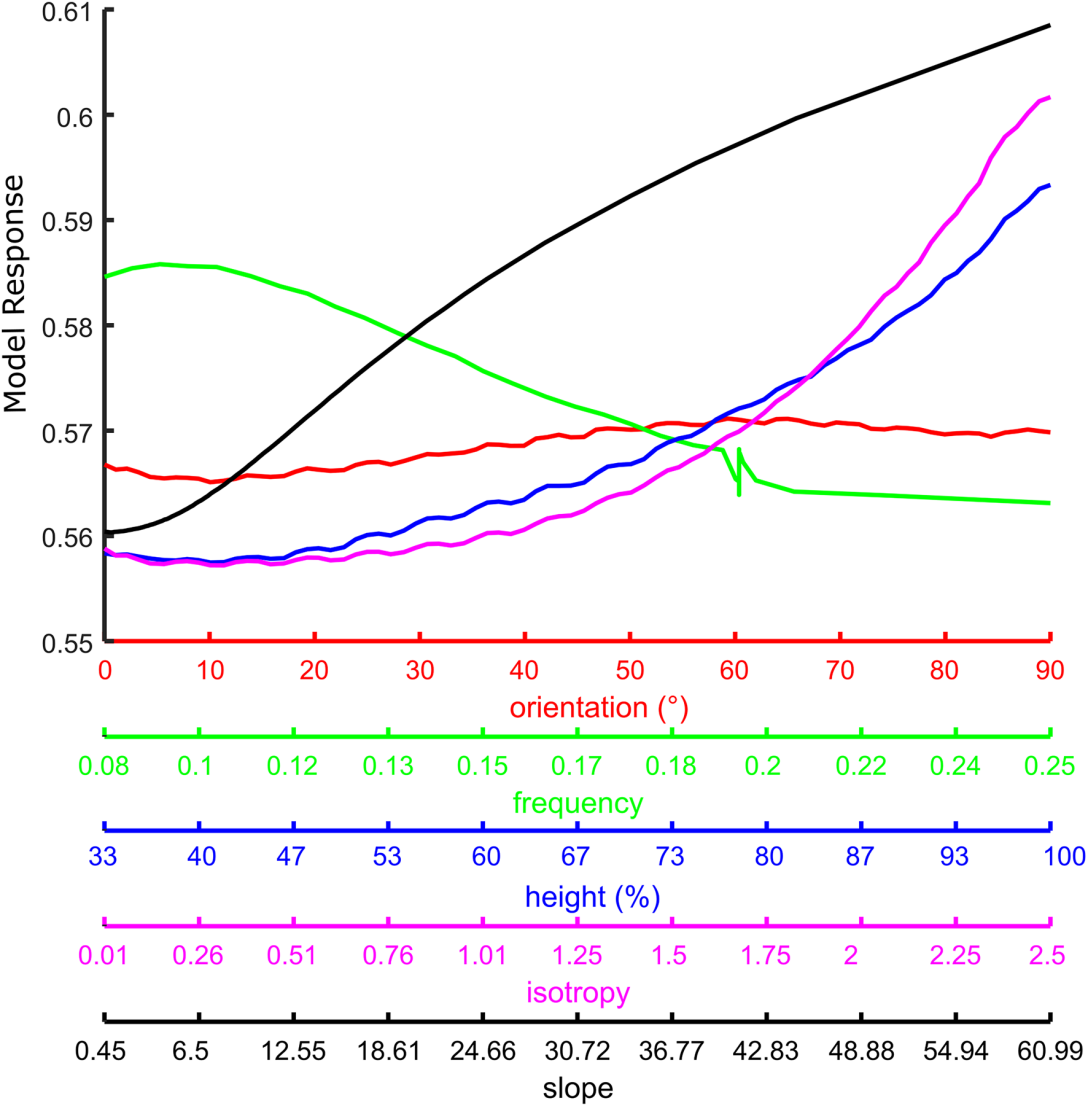
Reverse correlation. Model response on the *y*-axis, features on the *x*-axes: Height in blue, Orientation in red, Frequency in green and Anisotropy in magenta, slope in black.

Figure 8 shows that high model response implies high values in *Height* and *Anisotropy*, as observed with the previous analysis. Also, the top model’s response corresponds to rather low *Frequency,* consistent with the spatial tuning observed with the previous analysis. Model response and orientation do not seem to correlate, as orientation is stable at middle range for all model responses. This is not surprising as the model seems to be tuned to both vertical and horizontal angles.

Previous research shows that when exploring an object’s shape with the sense of touch, force cues elicited by the surface slope (i.e. resistance felt by the finger when sliding over a surface elevation, being higher with higher slope) are an important cue to shape perception^17,18^). Indeed it was shown that Gaussian profiles of the same height are haptically better detected at a smaller width, where they also have a higher slope^19,20^. Thus it could be expected that high surface slope would be a salient feature in haptic perception. We defined slope as gradient magnitude, as approximated by a Sobel Operator. Indeed, we found high model response with high surface slope (Fig. 8).

## Discussion

Haptic exploration of objects and materials usually consists of stereotypical *Exploratory Procedures* optimally chosen to extract certain information^2,3^. Also in haptic search systematic movements are observed^4–6^. Consistent with these findings, we found that when exploring unstructured surface reliefs freely or to decide whether two surface reliefs are same or different, participants’ exploratory behavior was partially stimulus independent (high predictive power of *Average Touch*). Also in eye-movements strong stimulus independent biases were observed^21^ such as the tendency to fixate in the center of the visual stimulus (*center bias*). Indeed, these tendencies are usually included in the currently best performing models to predict gaze location^22^. As we were interested in the relationship between haptic touch and local stimulus features, we did not provide the model with explicit information about stimulus independent behavior (e.g. touch duration averaged across different stimuli). Furthermore, we used random cropping for data-augmentation (see methods section), so that the model could never be presented with the full spatial distribution of touch duration in the training phase, thus promoting learning based on local features. However, our model learned, to a small extent, such stimulus independent tendencies, as it was performing above chance when predicting touch duration measured for a certain stimulus, based on the surface relief of different stimuli. Importantly, the difference between the stimulus independent performance and the stimulus dependent performance shows that haptic exploration is partially determined by local physical properties of the stimulus.

So far, only global rather than local effects of physical object properties on haptic exploration were observed and only for rather uniform materials such as velvet, fur, sand or elastomers. The choice of *Exploratory Procedures* was shown not to depend solely on the task but also on the global physical properties of the explored object^23^. For instance humans tend to spontaneously rub furry materials while they prefer to indent deformable objects such as playdouh or sponge when judging softness. Also parameters of *Exploratory Procedures* such as finger indentation force when judging an object’s softness^24^ or the scanning velocity of the hand in a roughness judgment task^25–27^ were shown to depend on the global physical properties of the explored object. In contrast, in our experiments participants explored objects with non-uniform surfaces. We showed that we can predict the locations participants would preferentially touch from local physical features of the surface. Thus, our findings indicate that haptic exploratory behavior is also affected by local physical features when the surface of the object is non-uniform.

In our first attempt to understand whether predicting touch behavior from local physical features is at all possible, we used a simple linear regression model^9^. Provided with the generative features of the stimuli, more precisely spatial distributions of height, isotropy, orientation gradient and frequency gradient, the model could predict local touch duration better than chance. However, we could use only a limited number of predictors (features we used to generate the stimuli) and any non-monotonic relationship such as a tuning function could not be captured. Crucially, the linear model can not be applied to different stimuli, such as natural surfaces, which were not generated based on the same predefined specific set of features. Further more, its informative value concerning salient features for humans is limited to interpreting the impact of these features. Here we present a more general saliency model, which allows to predict saliency for any stimulus.

Investigating the model’s responses to different physical properties of the input stimuli allows to infer potential salient features for human participants. This is because the model has learned the association between stimulus properties and touch behavior, to some extent, and therefore salient features are likely represented by stimulus properties preferred by the *DNN model*. We found model preference for high slope. This finding suggests that when participants moved their finger over the surface relief and perceived quick changes in lateral force, they further explored these locations.

For spatial frequency, we found a tuning around 0.2–0.3 cycles per mm. This frequency is close to the one found to be perceived as the most rough when actively exploring 3D printed gratings^28^. It was shown with a haptic search task that rough patches pop-out among smooth ones^29^, indicating that rough stimuli are salient relative to smooth ones. Our results suggest that roughness is a salient object feature also during haptic exploration.

We found further that high anisotropy and elevated modulations seem to be salient features, in agreement with our preliminary findings^9^. Previously it was observed that humans spontaneously follow the contours of the object when exploring its shape^1,2^. Our results suggest that also when exploring an unknown unstructured surface relief, participants preferred to explore elevated anisotropic modulations (with the edges being an extreme of it). We speculate that in haptic exploration humans spontaneously focus on surface structures which are likely informative about the shape of objects.

The model exhibited a preference for cardinal directions. Cardinal orientations can be discriminated better in active exploration (*oblique effect*^30,31^). Also, for the haptic perception of force cues an oblique effect was shown^32^. Additionally, we previously found that humans tend to move parallel to the edges of the haptically explored stimulus^6^ thus these stimulus orientations more likely produce high force cues at the finger, which was found to be a salient feature.

One might doubt that the model tuning properties actually reflect human preferences, given that the models performance is not particularly high. However, high performance does not necessarily imply that the model reproduces operations implemented in the human brain. Networks which achieve human-level performance when classifying objects in natural scenes fail drastically with small distortions such as salt-and-pepper-noise^33^ or contrast manipulation^34^, indicating that a network with human-level performance, might perform very different operations. Crucially, lower performance networks may better correlate with human brain recordings and behavioral measurements^35^. However, there is no guarantee that our model’s tuning properties reflect human perceptual tuning, but this possibility is strengthened by the analogies with previous research. The model preferred stimulus features which were shown to pop out in haptic search or for which humans are more sensitive than others—thus features which are likely to be salient.

We found that the *DNN model* trained on the exploration behavior in the “same or different” task successfully predicted which stimulus locations would be touched when the same stimuli are explored freely without any task. Exploratory behavior could be also predicted in an ecologically more valid condition, in which participants freely explored natural surfaces such as cliff rocks or cracked earth. The finding that our saliency model generalizes to a new task and to new, natural stimuli, strengthens the idea that the exploration was at least partly determined by physical stimulus features independent of the task and the specific stimuli. It is indeed possible that the salient parts of the stimulus which participants spontaneously preferred to touch when freely exploring the stimulus were the ones they considered as the most distinctive parts in order to compare the stimulus to another one. Performance of the model was lower when tested on the other task, possibly because the model was trained on a different data set. However, it can also reflect task influences, which were extensively shown for visual saliency (for review see^36,37^). Thus, possibly larger task effects could be expected in comparison to another task e.g. roughness judgment.

We considered local increase of touch duration with the index and middle fingers to reflect best detailed analysis and be thus best related to saliency similar to vision where saliency is reflected by fixation density. In a previous study we showed that humans express foveation-like behavior in haptic search, i.e. they first detect potential targets with any of the fingers and then analyze them with the index or middle finger^7,8^. Only these two fingers slow down during contact with potential targets and move within a small area. In fact, our model, trained on the index and middle finger, is poorer in predicting touch duration of the other fingers (AUC = 0.55, 0.49, 0.48, averaged over selection criterion, for the ring finger, little finger and thumb, respectively), corroborating the hypothesis of the specialized behavior of the middle and index finger. Noteworthy, if each next salient location has to be first available to the periphery, with large enough stimuli the scan-path should be predictable. In our analyses we neglected the dynamic aspects of explorations, i.e. we did not consider previous touch behavior as a predictor. Future research may exploit dynamic aspects of touching behavior to improve predictions and better understand haptic saliency, as incorporating sequential aspects of fixation behavior could improve visual saliency models^38^. Nonetheless, the model presented here does not include time and only predicts salience over space.

We used VQ-VAE as one of the numerous possibilities of deep networks which could learn the correspondence between local stimulus features and touch behavior. The simpler choice of a “vanilla” autoencoder (designed with a compression ratio comparable to the VQ-VAE), yields substantially lower performance (Supplementary Fig. S1). This is shown by an analogous ROC analysis, yielding AUC = 0.58 (averaged across selection criteria). Furthermore, stimulus dependent performance of the “vanilla” autoencoder is only marginally higher than stimulus independent (i.e. AUC = 0.57). This confirms our choice of the VQ-VAE.

Although our results also confirm the importance of stereotypical exploration movements, as indicated by *Average Touch*, we could demonstrate that part of exploratory touch behavior can be predicted based on local physical stimulus features. Form the preferences of the *DNN model*, we could infer that humans tend to explore more in detail regions of the haptic textures characterized by specific local properties: Edge-like structures (elevated and anisotropic), with a preference for vertical and horizontal patterns, rough regions, and surface modulations which elicit quick changes in lateral force. However, future research is necessary to test these preferences empirically and other ecologically valid conditions (i.e. real objects), as our model can generate haptic saliency predictions for any rigid surface. Unraveling salient haptic features could help designing intuitive user interfaces, haptic guidance aids but also improve the usability design of every-day objects.

## Methods

### Participants

12 students (9 females, average age 24.1, range 19–27 years) participated in Experiment 1. In Experiment 2, there were 10 participants (7 female, average age 23.3, range 20–28 years). And in Experiment 3 there were 8 participants (6 females, average age 22.4, range 19–25). All participants were volunteers, naive to the purpose of the experiment and were reimbursed for their participation (8€/h). All participants were right-handed and did not report any sensory or motor impairment at the right hand. The study was approved by the local ethics committee LEK FB06 at Giessen University and was in line with the declaration of Helsinki from 2008. Written informed consent was obtained from each participant.

### Stimuli

The haptic stimuli were printed using a 3D printer (Object30Pro, Stratasys, material VeroClear, nominal resolution 600 to 1600 dpi). They were first generated as 2D-images, and then translated into printable 3D models (Fig. 1B) using the OpenSCAD *surface()* function. The stimuli in Experiment 1 & 2 were 13.97 × 13.97 × 0.5 cm. The upper surface of the stimuli was defined to spatially vary in four features: height (vertical depth), spatial frequency, orientation and anisotropy. The spatial distributions of the features were defined by different 2D pink-noise distributions (same size as the stimulus, [0 1] range) coding for feature meaningful values within predefined ranges (Fig. 1C, bottom row). For instance in the frequency map, high values referred to high spatial frequency whereas in the orientation map high values coded for vertical orientation. The feature maps contained only spatial frequencies whose spatial period was lower than the average size of a fingertip (1.27 cm) ensuring that changes of feature values could be detected by the fingertips. To combine the features in one stimulus we used uniform white noise [0 1] images (base). For every pixel we filtered the base in the frequency domain using a filter with parameters *a*_*i*_, *φ*_*xyi*_ and *σ*_*ai*_ taken from the orientation, frequency and anisotropy feature maps respectively at the same spatial location defined by the following equation:

1 $${F}_{i}\left({\varphi }_{x},{\varphi }_{y}\right)={e}^{\left(\frac{-{\left(\sqrt{{\varphi }_{x}^{2}+{\varphi }_{y}^{2}}-{\varphi }_{xyi}\right)}^{2}}{{\sigma }_{f}^{2}}\right)}{e}^{\left(-{\sigma }_{ai}cos\left(2\left({a}_{xy}-{a}_{i}\right)\right)\right)}$$

*F*_*i*_ denotes the filter for the pixel *i,* defined as a function of the two dimensional Fourier frequency vector (*φ*_*x*_*,φ*_*y*_*).* It is a product of two Gaussians resulting in an oriented band pass filter. The first defines a Gaussian ring (of a fixed width *σ*_*f*_ = 0.01 cycles per image) centered at the origin of frequency space, limiting frequencies to a band centered at the frequency around the frequency *φ*_*xyi*_ (ranging from 0.08 to 0.50 mm^−1^) determined by the frequency map at pixel *i.* The second term is a circular Gaussian with a mean at angle *a*_*i*_ (ranging from 0° to 90°) as prescribed by the orientation map at pixel *i* and with a width *σ*_*ai*_ (ranging from 0 = completely isotropic to 1.8 radiants, most anisotropic, higher values do not produce appreciable changes in the stimuli at our resolution), determining the range of orientations and by this the degree of anisotropy according to the anisotropy map at pixel *i. a*_*xy*_, denotes the angle in the polar representation of *φ*_*x*_*, φ*_*y*_*.* Supplementary Fig. S2 shows how different feature parameters affect the filter in the frequency space. The resulting image was then multiplied with the height feature map and pixel brightness was interpreted as depth ranging from 0.1 to 0.3 cm. To each stimulus we added a 0.2 mm thick base to make it more stable. We produced 60 different maps by using 60 different seeds of pseudorandom number generator of MATLAB (The MathWorks, Inc., Natick, MA). All the maps and their feature distributions are shown in Supplementary Fig. S3.

The stimuli in Experiment 3 were generated from 3D scans available at textures.com. We printed 15 × 15 cm big cutouts from the following four surfaces: *Mud Cracked 1* (0.9 cm), *Dusty Soil* (0.7 cm)*, Cliff Layered* (1 cm) and *Pebbles 1* (0.8 cm)*.* The maximum height is specified in brackets. Selection criteria were reasonable resolution (< 2 cm/pixel) and local variability. The cutout was randomly selected from the entire 3D scan.

### Setup

Participants sat at a table together with the experimenter at their right side, who positioned a stimulus in front of them in each trial according to the instructions displayed at a monitor. The center of the stimulus was located approximately 15 cm away from the body and approximately aligned with the sagittal body plane (Fig. 1A). To stabilize the stimuli we attached stimulus supports at the four corners (Fig. 1A). The experiment was controlled by a computer program in MATLAB (MathWorks, Natick, MA, USA). The position of each finger of the right hand was tracked at 50 Hz in 3D space with the Zebris ultrasound system (Zebris Medical GmbH, Isny). The nominal resolution of the system is under 0.1 mm and the nominal accuracy, at the measurement distance used in the setup (around 35 cm), is under 1 mm. The markers were attached to the fingernails with adhesive pads (UHU patafix, Bolton Adhesives).

### Procedure

In all experiments the experimental session for each participant was preceded by a calibration in which the position of the right index finger was measured at the four corners of the stimulus. Each of the stimulus support corners contained in the middle a little cone (1.5 × 0.75 mm, calibration bump in Fig. 1A). Participants positioned the right index finger sequentially on each of these cones where the position was recorded for 3 s. The recorded positions for each corner were averaged and used to define a projective transformation to map touched positions onto the horizontal stimulus plane. The same transformation was used for the other fingers. During the experimental session participant were blind-folded. Each stimulus was presented twice, resulting in 120 trials in total in Experiment 1 & 2 and 8 trials in Experiment 3. The order of the trials was randomized.

In Experiment 1 participants were instructed to explore the first stimulus in order to compare it to another one presented subsequently. However, only in 30% of the trials (unpredictable for participants at the point of the exploration of the first stimulus) a comparison stimulus was actually presented, to save time. Participants were told that some trials stop after the exploration of the first stimulus, and no comparison needs to be performed. Before the beginning of each trial participants held their hand at the waiting position, which was defined by a little finger holder (3 × 2 × 0.5 cm) for the middle finger containing a central cylindrical cavity (Fig. 1A). Each trial began with a trial-start signal tone, played after the experimenter placed the first stimulus in front of the participant, informing them that they could start the exploration. Once they felt they sufficiently explored the first stimulus they returned the hand to the waiting position. Exploration was not limited in time. In the case there was a comparison, the experimenter placed a comparison stimulus in front of them and a different signal tone was played. In half of these trials it was the same stimulus and in the other half a different one (randomly chosen from the remaining stimuli) and participants reported their decision (same or different) by pressing two different keyboard buttons. In case there was no comparison, the trial-start signal was played and a new trial began. Finger positions were only recorded during the exploration of the first stimulus. Experiment 1 was completed on average within 1.5 h.

In Experiments 2 and 3 participants were instructed to explore the stimulus freely (*“Please try to use your sense of touch to understand what kind of object is in front of you.”*). Participants placed their hand at the waiting position once they sufficiently explored the stimulus and a new trial began. Experiment 2 was completed on average within 1 h and Experiment 3 on average within 15 min.

### Data analysis

Exploratory behavior was quantified as touch duration at each position of the stimulus. We computed touch duration only for the index and the middle finger, because these fingers are mostly involved in fine analysis of the stimulus^6–8^. For every trial and each sample, the pixels around the current positions of the index and middle fingers within the approximate area of a fingertip (1.27 cm) were considered as touched (1) and the other pixels as not touched (0). For each pixel the sum across samples was then divided by the sampling frequency (50 Hz) to compute touch duration. Touch duration was then normalized to 1 for each trial (to avoid that some trials influence the model training more than others) and saved as 8 bits images, which is the format for the model’s output and predictions. We expressed model’s predictions as percentage of the maximum value (i.e. 255), for data analysis and visualization. For the ROC curve, the true positive rate was computed as the number of high touch duration pixels (Fig. 4C, red area) whose corresponding predicted touch duration was higher than a given criterion divided by the total number of high duration pixels. The false positive rate was computed as the number of low touch duration pixels whose corresponding predicted touch duration exceeded the criterion, divided by the total number of low duration pixels. The ROC curve is obtained by computing true positive rate and false positive rate for different criteria corresponding to all the values of the model’s prediction image, so that the AUC is criterion-independent measure. The same logics is followed for each prediction, independent of its unit of measure.

### DNN model

We trained all networks from scratch without using pre-trained weights, on a single GPU with batch size of 64. The loss function of trained VQ-VAEs is defined as follows,

2 $$L=logp\left(x\vee {z}_{q}\left(x\right)\right)+{\Vert sg\left[{z}_{e}\left(x\right)\right]-e\Vert }_{2}^{2}+\beta {\Vert {z}_{e}\left(x\right)-sg\left[e\right]\Vert }_{2}^{2}$$

where *sg* denotes the stop gradient computation that is defined as the identity during the forward-propagation, and with zero partial derivatives during the back-propagation to refrain its update. The first term in Eq. (2) corresponds to the reconstruction loss incorporating both encoder and decoder; the second term updates the embedding vectors; and the third term, referred to as the commitment loss, harmonizing the encoder and embedding vectors. The parameter $\beta \in R$ is set to 0.5 in all our experiments.

In this work, we set both *K* and *D* to 64 (size of the embedding space). The spatial size of surface relief map and touch duration map is 330 × 330. We trained our models with Adam optimizer^39^ ($lr=2\times {10}^{-4}$) for 100 epochs. In order to augment the data, during the training we randomly cropped a 260 × 260 portion of surface relief map and touch duration map. The surface relief maps and touch duration maps have been stored in 8 bits PNG images that prior to their input to the network were normalized to the range between 0 and 1.

### Virtual Textures to correlate with model’s response

In order to correlate features and model’s responses, 2200 virtual surface reliefs were generated with the same algorithm and range of parameters as the ones used for the experimental stimuli. Different seeds of MATLAB (MathWorks, Natick, MA, USA) pseudorandom number generator ensured that the textures were different. To generate the uniform textures, the parameters were not varying over space, i.e. each of the four features corresponded to a single value for the whole stimulus. Local variations were due to the base white noise image. We systematically varied features values, for all the combinations of 15 values per feature. *Height* varied linearly between 7% and 100%, *Anisotropy* from 0 to π and *Orientation* from 0° to 90°. *Frequency* varied exponentially from 0.006 to 0.5 mm^-1^. The stimuli were 256 × 256 pixels, consistent with the model’s setting.

## Supplementary Information

Supplementary Information.

## Data Availability

Behavioral data of individual participants from all experiments and the code for the DNN model can be downloaded in our GitHub repository https://github.com/ArashAkbarinia/DeepTouch.

## References

[1] Gibson J (1962). Observations on active touch. Psychol. Rev..

[2] Lederman SJ, Klatzky RL (1987). Hand movement: A window into haptic object recognition. Cogn. Psychol..

[3] Cavdan, M., Doerschner, K., & Drewing, K. The many dimensions underlying perceived softness: How exploratory procedures are influenced by material and the perceptual task. *2019 IEEE World Haptics Conference (WHC)* 437–442 (2019).

[4] Morash, V. S. Detection radius modulates systematic strategies in unstructured haptic search. *2015 IEEE World Haptics Conference (WHC)* 1–6 (2015).

[5] Morash VS (2016). Systematic movements in Haptic Search: Spirals, zigzags, and parallel sweeps. IEEE Trans. Haptics.

[6] Metzger, A., Toscani, M., Valsecchi, M., & Drewing, K. Target search and inspection strategies in haptic search. *IEEE Transactions on Haptics*, under review (2021). 10.1109/TOH.2021.3076847 33929965

[7] Metzger, A., Toscani, M., Valsecchi, M., & Drewing, K. Dynamics of exploration in haptic search. *2019 IEEE World Haptics Conference (WHC)* 277–282 (2019).

[8] Metzger, A., Toscani, M., Valsecchi, M., & Drewing, K. Foveation-like behavior in human haptic search. *In preparation* (2021)*.*

[9] Metzger A, Toscani M, Valsecchi M, Drewing K, Prattichizzo D, Shinoda H, Tan H, Ruffaldi E, Frisoli A (2018). Haptic Saliency Model for Rigid Textured Surfaces. Haptics: Science, Technology, and Applications. EuroHaptics 2018. Lecture Notes in Computer Science.

[10] Hsiao S (2008). Central mechanisms of tactile shape perception. Curr. Opin. Neurobiol..

[11] Van Den Oord, A., & Vinyals, O. Neural discrete representation learning. *Advances in Neural Information Processing Systems* 6306–6315 (2017).

[12] Yang, J., Price, B., Cohen, S., Lee, H., & Yang, M. H. Object contour detection with a fully convolutional encoder-decoder network. In *Proceedings of the IEEE Conference on Computer Vision and Pattern Recognition *193–202 (2016).

[13] Alex V, Vaidhya K, Thirunavukkarasu S, Kesavadas C, Krishnamurthi G (2017). Semisupervised learning using denoising autoencoders for brain lesion detection and segmentation. J. Med. Imaging.

[14] Han K, Wen H, Shi J, Lu KH, Zhang Y, Fu D, Liu Z (2019). Variational autoencoder: An unsupervised model for encoding and decoding fMRI activity in visual cortex. NeuroImage.

[15] Luo W, Li J, Yang J, Xu W, Zhang J (2017). Convolutional sparse autoencoders for image classification. IEEE Trans. Neural Netw. Learn. Syst..

[16] Green DM, Swets JA (1966). Signal Detection Theory and Psychophysics.

[17] Robles-De-La-Torre G, Hayward V (2001). Force can overcome object geometry in the perception of shape through active touch. Nature.

[18] Drewing K, Ernst MO (2006). Integration of force and position cues for shape perception through active touch. Brain Res..

[19] Louw S, Kappers AM, Koenderink JJ (2000). Haptic detection thresholds of Gaussian profiles over the whole range of spatial scales. Exp. Brain Res..

[20] Nefs HT, Kappers AML, Koenderink JJ (2001). Amplitude and spatial-period discrimination in sinusoidal gratings by dynamic touch. Perception.

[21] Tatler BW, Vincent BT, Vincent T (2009). The prominence of behavioural biases in eye guidance. Vis. Cogn..

[22] Kümmerer, M., Wallis, T. S. A., & Bethge, M. DeepGaze II: Reading fixations from deep features trained on object recognition. Preprint at https://arxiv.org/abs/1610.01563 (2016).

[23] Dovencioglu D, Doerschner K, Drewing K (2019). Aspects of material softness in active touch. PERCEPTION.

[24] Lezkan A, Metzger A, Drewing K (2018). Active haptic exploration of softness: Indentation force is systematically related to prediction, sensation and motivation. Front. Integr. Neurosci..

[25] Callier T, Saal HP, Davis-Berg EC, Bensmaia SJ (2015). Kinematics of unconstrained tactile texture exploration. J. Neurophysiol..

[26] Gamzu E, Ahissar E (2001). Importance of temporal cues for tactile spatial-frequency discrimination. J. Neurosci..

[27] Tanaka Y, Bergmann Tiest WM, Kappers AML, Sano A (2014). Contact force and scanning velocity during active roughness perception. PLoS ONE.

[28] Drewing K, Prattichizzo D, Shinoda H, Tan H, Ruffaldi E, Frisoli A (2018). Judged Roughness as a Function of Groove Frequency and Groove Width in 3D-Printed Gratings. Haptics: Science, Technology, and Applications EuroHaptics 2018 Lecture Notes in Computer Science.

[29] Plaisier MA, Bergmann Tiest WM, Kappers AML (2008). Haptic pop-out in a hand sweep. Acta Physiol. (Oxf).

[30] Lechelt EC, Eliuk J, Tanne G (1976). Perceptual orientational asymmetries: A comparison of visual and haptic space. Percept. Psychophys..

[31] Gentaz E, Baud-Bovy G, Luyat M (2008). The haptic perception of spatial orientations. Exp. Brain Res..

[32] Yang, X. D., Bischof, W. F., & Boulanger, P. Perception of haptic force magnitude during hand movements. *Proceedingsof IEEE International Conference on Robotics and Automation* 2061–2066 (2008).

[33] Geirhos R, Temme CR, Rauber J, Schütt HH, Bethge M, Wichmann FA (2018). Generalisation in humans and deep neural networks. Adv. Neural Inf. Process. Syst..

[34] Akbarinia A, Gil-Rodríguez R (2020). Deciphering image contrast in object classification deep networks. Vis. Res..

[35] Kubilius J (2019). Brain-like object recognition with high-performing Shallow recurrent ANNs. Adv. Neural Inf. Process. Syst..

[36] Hayhoe M, Ballard D (2005). Eye movements in natural behavior. Trends Cogn. Sci..

[37] Land MF (2006). Eye movements and the control of actions in everyday life. Prog. Retinal Eye Res..

[38] Foulsham T, Underwood G (2008). What can saliency models predict about eye movements? Spatial and sequential aspects of fixations during encoding and recognition. J. Vis..

[39] Kingma, D. P., & Ba, J. Adam: A method for stochastic optimization. Preprint at https://arxiv.org/abs/1412.6980 (2014).

