# Supplementary material for: Deep neural network model of haptic saliency
Source: Sci Rep. 2021 Jan 14;11:1395. doi: 10.1038/s41598-020-80675-6 (PMC7809404; doi:10.1038/s41598-020-80675-6)
Supplement: Supplementary file 1 — Supplementary Information. [file 41598_2020_80675_MOESM1_ESM.pdf]

Deep neural network model of haptic saliency

Anna Metzger, Matteo Toscani, Arash Akbarinia, Matteo Valsecchi & Knut Drewing

Supplementary Information

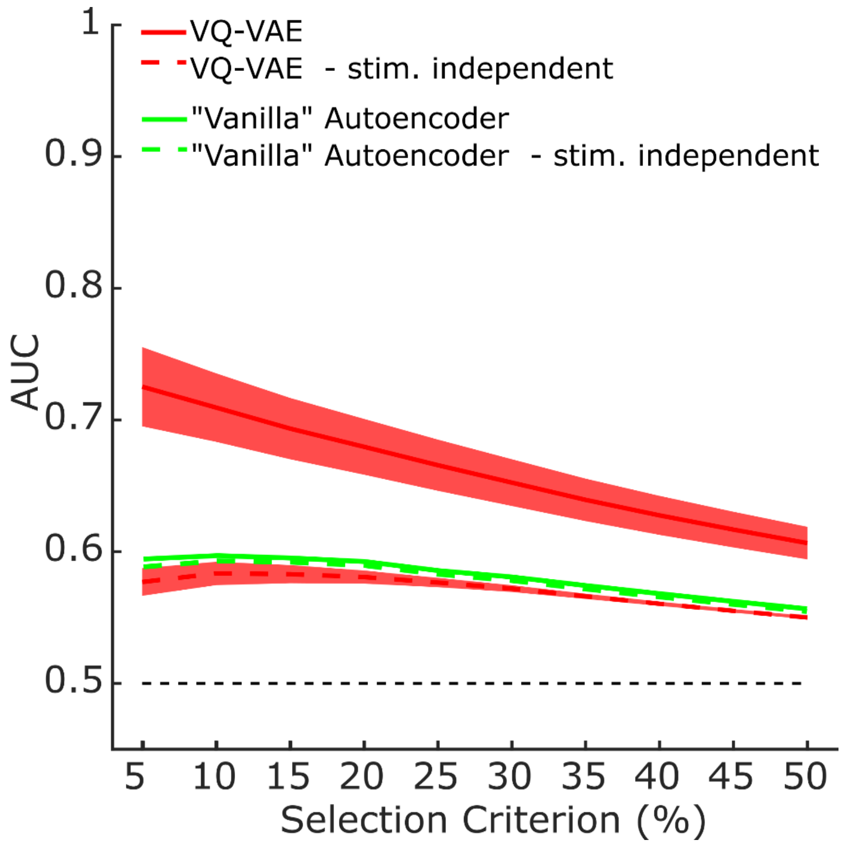

Supplementary Figure S1: ROC analysis for the "Vanilla" and variational autoencoders. AUC (y-axis) computed with different selection criteria (x-axis). AUCs for the different models are depicted with different colors, as indicated in the legend. Continuous lines represent the performance based on predictions computed for the stimuli that were actually explored in every trial. Dashed lines represent the stimulus independent AUCs. AUCs are averaged across participants; the colored areas represent the standard error of the mean.

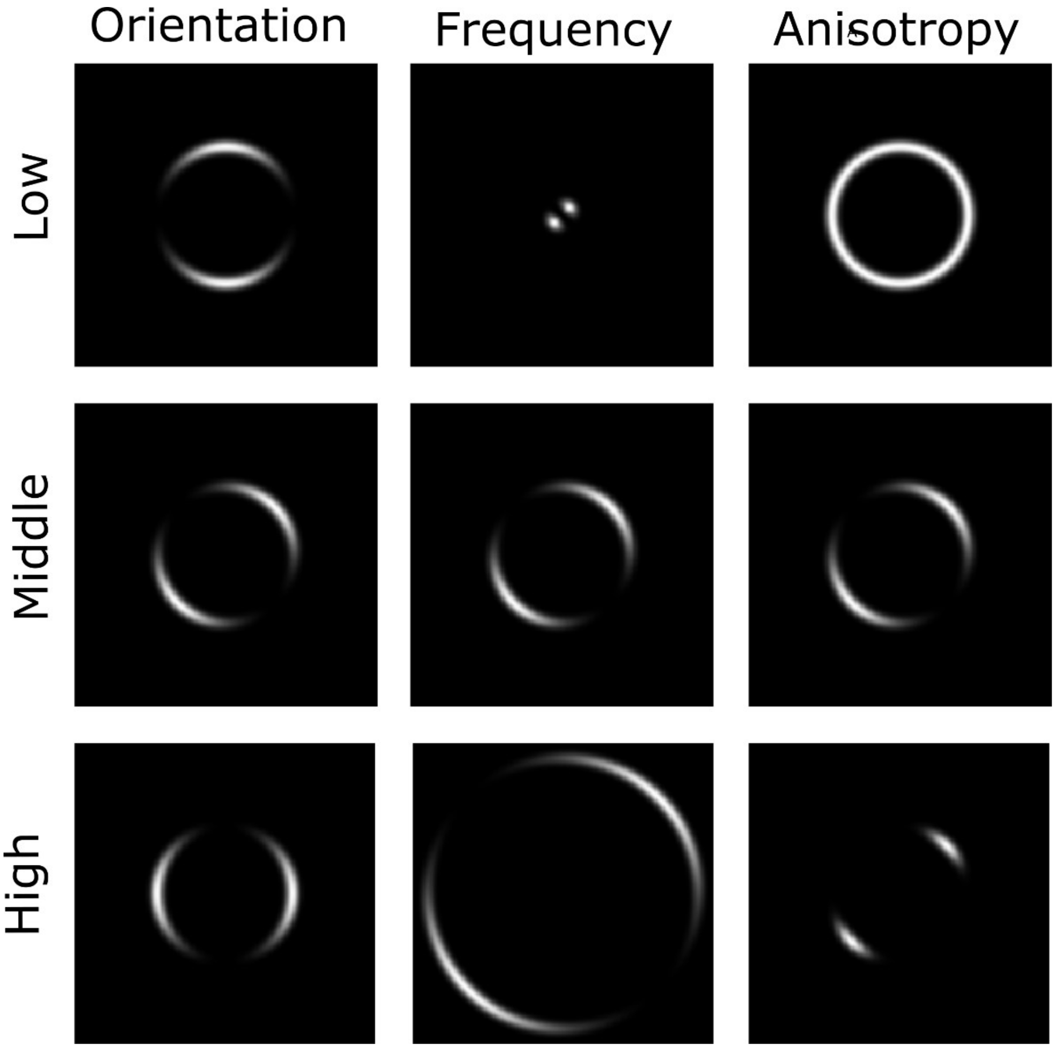

Supplementary Figure S2: Examples of filters in the frequency domain used to create the stimuli for Experiment 1 & 2. Each column shows how three levels of parameters (Low, Middle and High) of each feature (Orientation, Frequency and Anisotropy) affected the filter in the frequency domain.

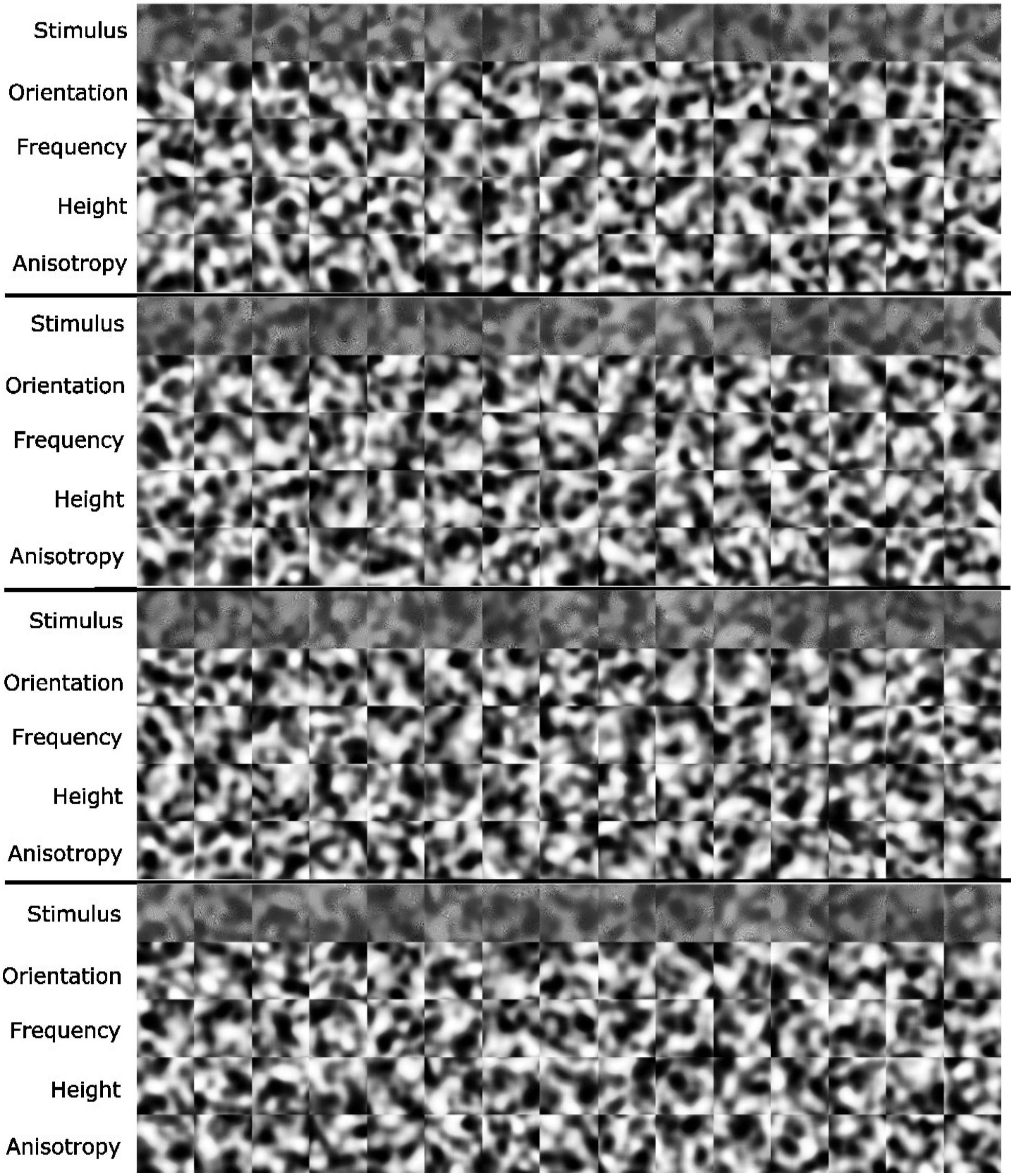

Supplementary Figure S3: All stimuli and their feature distributions used in Experiments 1 & 2.
